# CRISPR/Cas9-mediated *VvPR4b* editing decreases downy mildew resistance in grapevine (*Vitis vinifera* L.)

**DOI:** 10.1038/s41438-020-00371-4

**Published:** 2020-09-01

**Authors:** Meng-Yuan Li, Yun-Tong Jiao, Yu-Ting Wang, Na Zhang, Bian-Bian Wang, Rui-Qi Liu, Xiao Yin, Yan Xu, Guo-Tian Liu

**Affiliations:** 1 State Key Laboratory of Crop Stress Biology in Arid Areas, College of Horticulture, Northwest A&F University, Yangling, Shaanxi 712100 China; 2 Key Laboratory of Horticultural Plant Biology and Germplasm Innovation in Northwest China, Ministry of Agriculture, Northwest A&F University, Yangling, Shaanxi 712100 China

**Keywords:** Transgenic plants, Genetic engineering, Molecular engineering in plants

## Abstract

Downy mildew of grapevine (*Vitis vinifera* L.), caused by the oomycete pathogen *Plasmopara viticola*, is one of the most serious concerns for grape production worldwide. It has been widely reported that the pathogenesis-related 4 (PR4) protein plays important roles in plant resistance to diseases. However, little is known about the role of PR4 in the defense of grapevine against *P. viticola*. In this study, we engineered loss-of-function mutations in the *VvPR4b* gene from the cultivar “Thompson Seedless” using the CRISPR/Cas9 system and evaluated the consequences for downy mildew resistance. Sequencing results showed that deletions were the main type of mutation introduced and that no off-target events occurred. Infection assays using leaf discs showed that, compared to wild-type plants, the *VvPR4b* knockout lines had increased susceptibility to *P. viticola*. This was accompanied by reduced accumulation of reactive oxygen species around stomata. Measurement of the relative genomic abundance of *P. viticola* in *VvPR4b* knockout lines also demonstrated that the mutants had increased susceptibility to the pathogen. Our results confirm that *VvPR4b* plays an active role in the defense of grapevine against downy mildew.

## Introduction

Grapevine is one of the most economically important fruit crops worldwide and is widely used for producing wine, juice, and dried and fresh fruit^1^. However, its yield and quality are adversely affected by abiotic and biotic factors. Grapevine downy mildew, caused by the oomycete *P. viticola* (Berk. et Curt.) Berlese and De Toni, is among the most serious fungal diseases of grapevine worldwide^2^. This pathogen infects green parts such as leaves, petioles, new shoots, and young fruit^3^.

Pathogenesis-related (PR) proteins comprise a large and heterogeneous class of plant disease defense proteins. Transgenic plants expressing PR proteins at high levels show significantly higher resistance to various pathogens. For example, in tobacco, constitutive expression of PR-1a confers increased resistance to two oomycete pathogens, namely, *Peronospora tabacina* and *Phytophthora parasitica var*. *nicotianae*^4^. In rice, ectopic expression of PR-5 increases resistance to rice sheath blight^5^. One of the most extensively studied PR proteins is PR4, which is a chitinase and chitin-binding protein^6^. PR4 genes have been cloned from several plants. Overexpression of β-1,3-glucanase and chitinase genes in ‘Crimson Seedless’ grapes conferred markedly improved resistance to downy mildew^7^. Previous studies have shown that PR4 additionally has DNase activity and inhibits fungal mycelium growth. However, although the PR4 proteins can be classified based on the presence (Class I) or absence (Class II) of an amino-terminal chitin-binding domain (ChtBD), this does not affect its fungal inhibition activity^8^. In jelly fig (*Ficus awkeotsang* Makino), deletion of ChtBD from the Class I FaPR-4 protein does not affect the DNase activity or antifungal function of this protein^9^. In grapevine, transgenic lines overexpressing PR4 showed increased resistance to powdery mildew^10^. Due to the inherent difficulties of transformation in grapevine and other perennial fruit crops, gene functional analyses have generally been limited to transient expression or transgene integration, and targeted gene knockout approaches have rarely been utilized. In grapevine, the most efficient approaches for *Agrobacterium*-mediated transformation have utilized proembryonic masses and somatic embryos (SEs)^11,12^.

The clustered regularly interspaced short palindromic repeats (CRISPR)/CRISPR-associated protein 9 (Cas9) system (CRISPR/Cas9 system), comprising the Cas9 endonuclease and guide RNA (gRNA), can be used to introduce heritable mutations at target sites^13^. The sgRNA comprises an ~20-bp target site sequence as well as a protospacer adjacent motif (PAM), required for the introduction of double-strand breaks (DSBs) by Cas9^14,15^. DSBs can be repaired by endogenous DNA-repair mechanisms, but this process frequently results in mutations.

The CRISPR/Cas9 system has been used for genome editing in many organisms^16^. Diverse CRISPR/Cas9 systems have exhibited high efficiency and specificity for gene editing in plants^17–19^. Chen et al. conveniently identified transgenic tobacco lines without using antibiotics, and these plants were confirmed to be allelic mutants^20^. In rice, the CRISPR/Cas9 system has been applied to quickly regenerate deletion mutants targeting the Tos17 retrotransposon, providing rapid breeding techniques^21^. Recently, high-efficiency targeted deletion and insertion mutagenesis in both grapevine and apple has been enabled by the direct introduction of purified CRISPR/Cas9 ribonucleoproteins into protoplasts^22^.

There have been very few reports on the successful use of the CRISPR/Cas9 system in perennial fruit crops such as grapevine. Ren et al. employed CRISPR/Cas9 in grapevine (cv. “Chardonnay”) suspension cells and plants to disrupt the *L-idonate dehydrogenase* gene related to tartaric acid^23^. Using two sgRNAs targeting distinct sites in the gene, they obtained a 100% mutation frequency in the transgenic cell mass (CM)^23^. In another study, Wang et al. employed the CRISPR/Cas9 system in the cultivar ‘Thompson Seedless’ to show that the transcription factor gene *VvWRKY52* is required for susceptibility to *Botrytis cinerea*^24^. They used gRNAs targeting four sites, and obtained 15 lines with biallelic mutations and 7 lines with monoallelic mutations. The editing events included both large (≥5 bp) and small deletions^24^. Nakajima et al. successfully mutagenized the grapevine *PHYTOENE DESATURASE* (*VvPDS*) gene using embryogenic calli^25^. In that experiment, loss of gene function was apparent in albino tissue^25^. They found that in the regenerated shoots, the ratio of albino cells in lower and older leaves was higher than that in new, young, higher leaves, possibly due to repeated induction of DSBs or the imprecision of DSB repair in the older leaves^25^. In another study targeting the *VvPDS* gene, Ren et al. surveyed the relationship between gene editing efficiency and the GC content of the sgRNA, kinds of materials used for genetic modification and expression level of SpCas9 in the transgenic CM^26^. Their research showed that sgRNA with a GC content of 65% had the highest CRISPR/Cas9 editing efficiency^26^. In addition, the expression of the SpCas9 protein correlated with the editing efficiency, but this effect was not as strong as that seen for the GC content^26^. Ren et al. successfully used CRISPR/Cas9 in grapevine to show that the *Carotenoid Cleavage Dioxygenase 8* (*VvCCD8*) gene is crucial for normal branching^27^.

Here, we utilized the CRISPR/Cas9 system to study the role of grapevine PR4 in the resistance to downy mildew.

## Results

### Cloning of *VvPR4b* from “Thompson Seedless”

The *VvPR4b* gene was cloned from “Thompson Seedless” using oligonucleotide primers (Table S1) designed to be specific for *VvPR4b* as annotated in the “Pinot Noir” reference genome (12×; http://www.genoscope.cns.fr). The *VvPR4b* open reading frame is 432 bp in length and encodes a 143-amino-acid product containing an N-terminal 21-amino-acid signal peptide and a conserved 125-amino-acid barley wound-induced (Barwin) domain. VvPR4b lacks an amino-terminal ChtBD and thus is a Class II PR4.

### Target selection and CRISPR/Cas9 vector construction

**Fig. 1:**
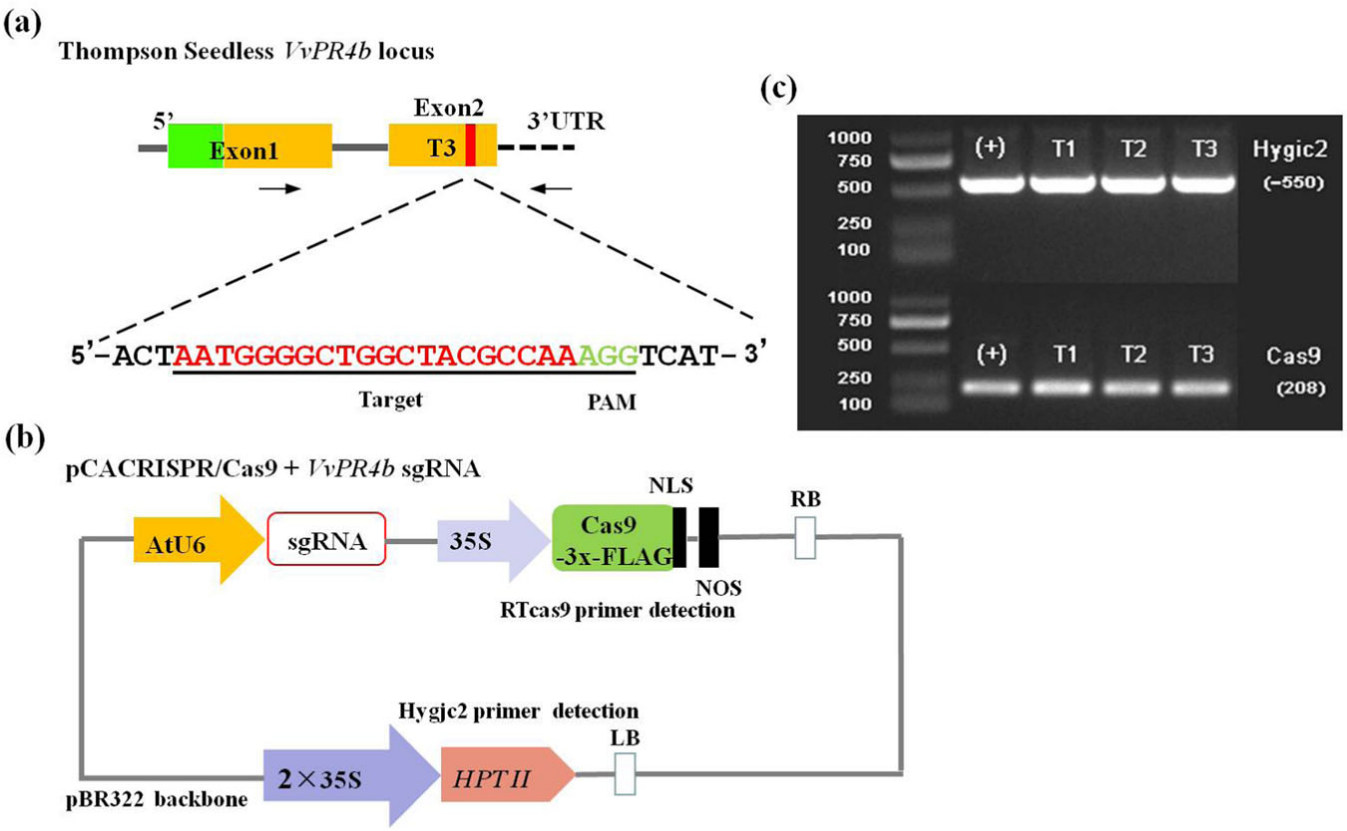
Construction of the *VvPR4b* knockout vector. **a** Schematic of the location of the T3 target site within exon 2 of the *VvPR4b* gene. Exons are shown as green and yellow blocks, where green indicates the signal peptide and yellow indicates the Barwin domain. Target site is indicated by vertical red bar. The expanded sequence for T3 includes the target sequence (red) and PAM trinucleotide (green). **b** Illustration of the *VvPR4b* knockout vector. **c** PCR amplification of two sequences (Hygjc2 and RTcas9) from the knockout vector

Three potential independent target gRNAs within the Barwin domain of *VvPR4b* were designed using the online tool CRISPR-P (http://cbi.hzau.edu.cn/cgi-bin/CRISPR) (Fig. 1a and Fig. S1). The target sequences were incorporated into the reverse primer (Table S1). Then, using the pCACRISPR/Cas9 expression vector as a template, forward primers were designed to amplify the AtU6 promoter, obtaining an expression cassette for AtU6 and VvPR4b-sgRNA (Table S1). Finally, the expression cassette was cloned into the linearized vector by homologous recombination to obtain the *VvPR4b* knockout vector, which contains a single guide RNA (Fig. 1b).

### Generation and identification of transgenic plants

**Fig. 2:**
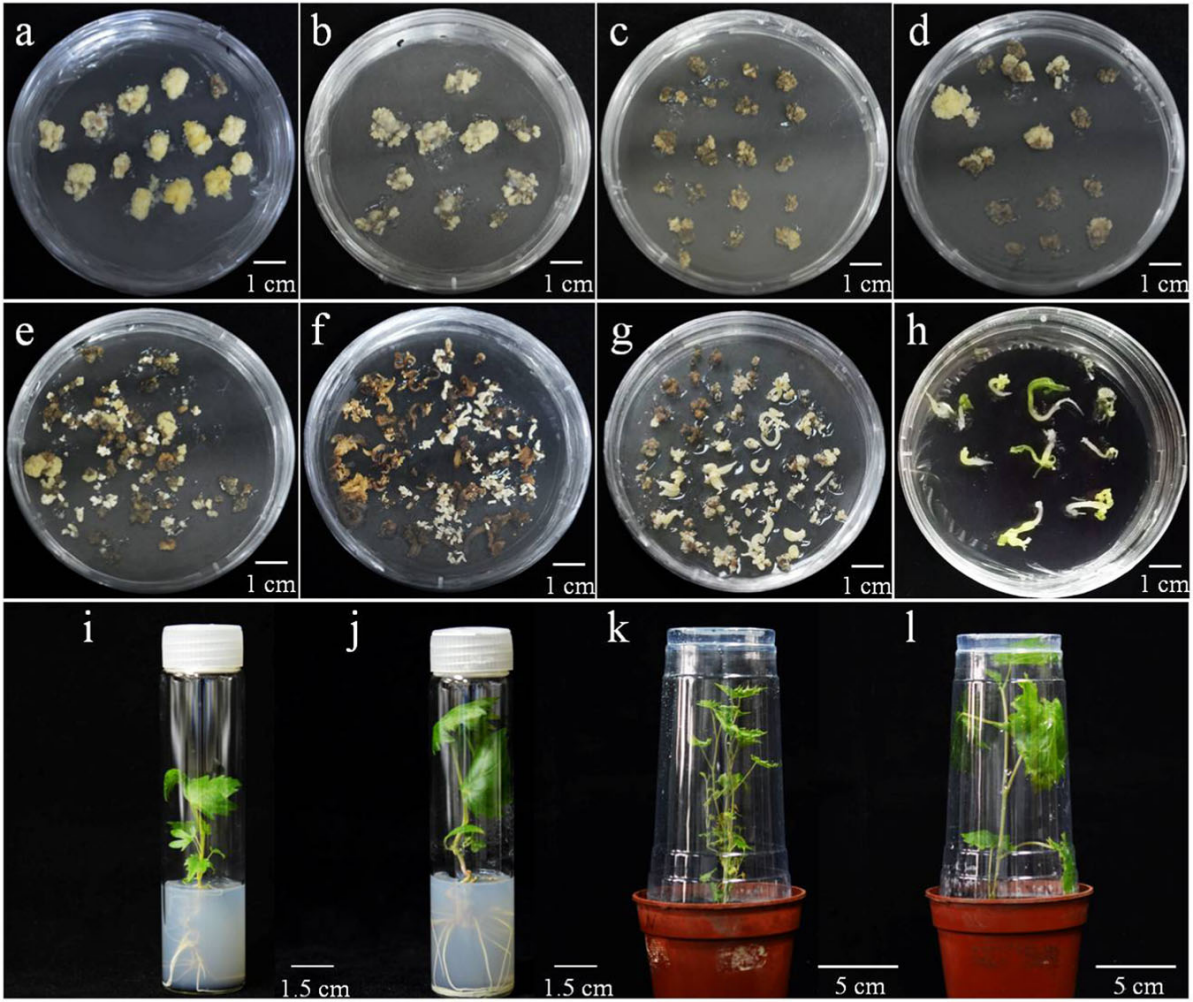
*Agrobacterium*-mediated regeneration of grapevine after genetic transformation. **a** Faint yellow secondary PEM cells used for transformation (X6 medium); (**b**) delayed screening and culture of transformed PEM cells after one month; (**c**, **d**) selection on screening medium containing antibiotics; (**e**–**g**) selection on differentiation medium (X3 screening medium); (**h**) cotyledons turned green and roots appeared after culture on X1.5 medium; (**i**, **j**) regenerated plantlets cultured on WPM medium; (**k**, **l**) regenerated seedlings were transplanted into soil and cultured in a controlled-environment chamber

Prior to transformation, proembryogenic mass (PEM) cells were cultured on X6 medium (Fig. 2a). After transformation, the PEM was transferred to delayed screening medium (Fig. 2b). Subsequently, the PEM was cultured on regeneration medium containing hygromycin for selection for ~3 months, during which time a faint yellow embryogenic callus differentiated (Fig. 2c, d). Hygromycin-resistant embryos were transferred to selection medium for further induction (Fig. 2e–g). The above processes were carried out in the dark at 26 °C. The induced cotyledons and hypocotyls were subcultured on X1.5 medium under light at 26 °C, during which time the cotyledons turned green (Fig. 2h). Each resistant shoot was transferred to woody plant medium (WPM) to induce seedling formation (Fig. 2i, j). Finally, seedlings were transplanted into nutrient substrate for further growth (Fig. 2k, l). In this study, the duration of the transformation cycle, from *Agrobacterium* inoculation to identification of mutant transgenic lines, was ~15 months.

**Fig. 3:**
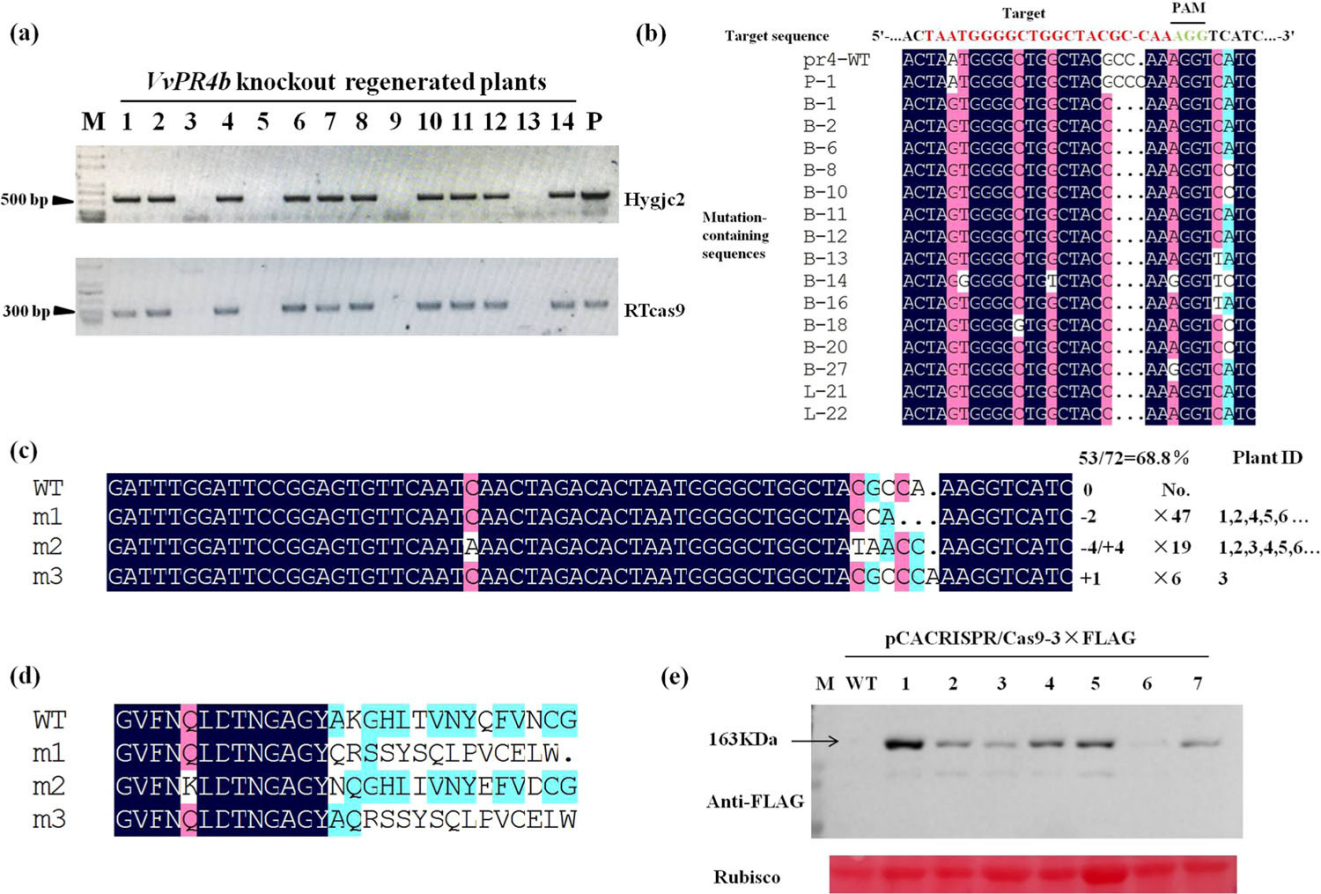
Detection and characterization of mutations within the *VvPR4b* gene in regenerated plants. **a** Identification of Hygjc2 and RTcas9 sequences in regenerated plants. Amplification in positive lines is expected to give products of 550 and 210bp, respectively. CRISPR-Cas9 plasmid DNA was used as a positive control (P). Lanes 1-14 represent distinct T0 regenerated plants tested. **b** Sequence of the T3 target site in regenerated plantlets. **c** The four distinct mutations observed in the T0 *VvPR4b* knockout lines. The sequence, number of regenerated clones containing the mutation, and identification number of regenerated plants are indicated. **d** Deduced amino acid sequences of the proteins predicted to be encoded by the mutant sequences shown in Fig. 3c. **e** Detection of Cas9 protein expression in the transgenic lines. Lanes 1–7 represent distinct transgenic plants. Protein extract from nontransgenic, wild-type plants (WT) was used as a negative control

To confirm the presence of the transgene in the regenerated plantlets, we extracted genomic DNA from leaves and performed PCR using oligonucleotide primers specific for vector sequences corresponding to RTcas9 and Hygjc2 of the 14 regenerated plants initially tested; 10 were found to contain both sequences (Fig. 3a).

For further study, we focused on transgenic lines targeting a site near the carboxyl-terminal end of the Barwin domain (T3 target site; Fig. 1a). We found that all 72 samples sequenced showed mutations within an ~5-base sequence at the T3 target sequence immediately upstream of the PAM site (Fig. 3b). The most frequently observed mutation (47 samples) was a dinucleotide deletion (CGCCA > CCA), resulting in a reading frame shift after Tyr127 in the encoded protein and in termination after an additional 16 amino acids (Fig. 3c, d). A complex mutation, CGCCA > TAACC, was also frequently observed (19 samples). This resulted in a nonconservative substitution of Asn-Gln for Ala-Lys129 in the encoded protein. In addition, all 19 samples containing the CGCCA > TAACC mutation additionally contained a C > A transversion 11 bases upstream of the 5′ end of the target sequence, converting Gln119 to Lys (Fig. 3b, c). Finally, we identified a small number of samples (6) containing a single nucleotide insertion of cytosine (CGCCA > CGCCCA), resulting in a reading frame switch after Ala128 in the encoded protein and termination after an additional 15 amino acids (Fig. 3c, d). The Cas9 protein encoded by the CRISPR/Cas9 vector contains a 3×-FLAG epitope tag for immunological detection (Fig. 1b). Western blotting of protein extracts from regenerated plants revealed an immunoreactive species of the size expected for the Cas9-3×-FLAG protein (163 kDa), which was absent in nontransgenic plants (Fig. 3e).

### Analysis of potential off-target mutations

Three potential off-target mutation sites were identified through CRISPR-P: (1) on chromosome 14 within an exon of the VIT_14s0081g00020 gene encoding a short, Barwin domain-containing protein; (2) an intergenic region of chromosome 16; and (3) on chromosome 11 within an intron of the VIT_11s0016g03800 gene encoding an uncharacterized protein (Table 1). We amplified each sequence from 10 independent samples using PCR and specific oligonucleotide primers (Table S1) and subjected the amplicons to sequencing. The results showed that there were no off-target events in this gene editing study (Table 1).

**Table 1:**
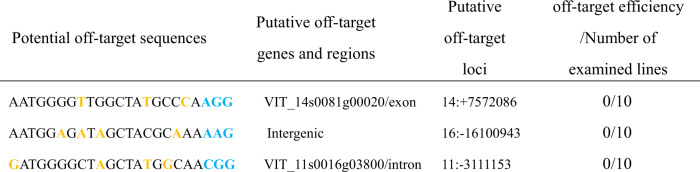
Analysis of potential off-target sites in transgenic plants

PAM sequences are indicated in blue. Mismatched nucleotides are marked in orange.

### Knockout of *VvPR4b* in “Thompson Seedless” decreases resistance to *P. viticola*

**Fig. 4:**
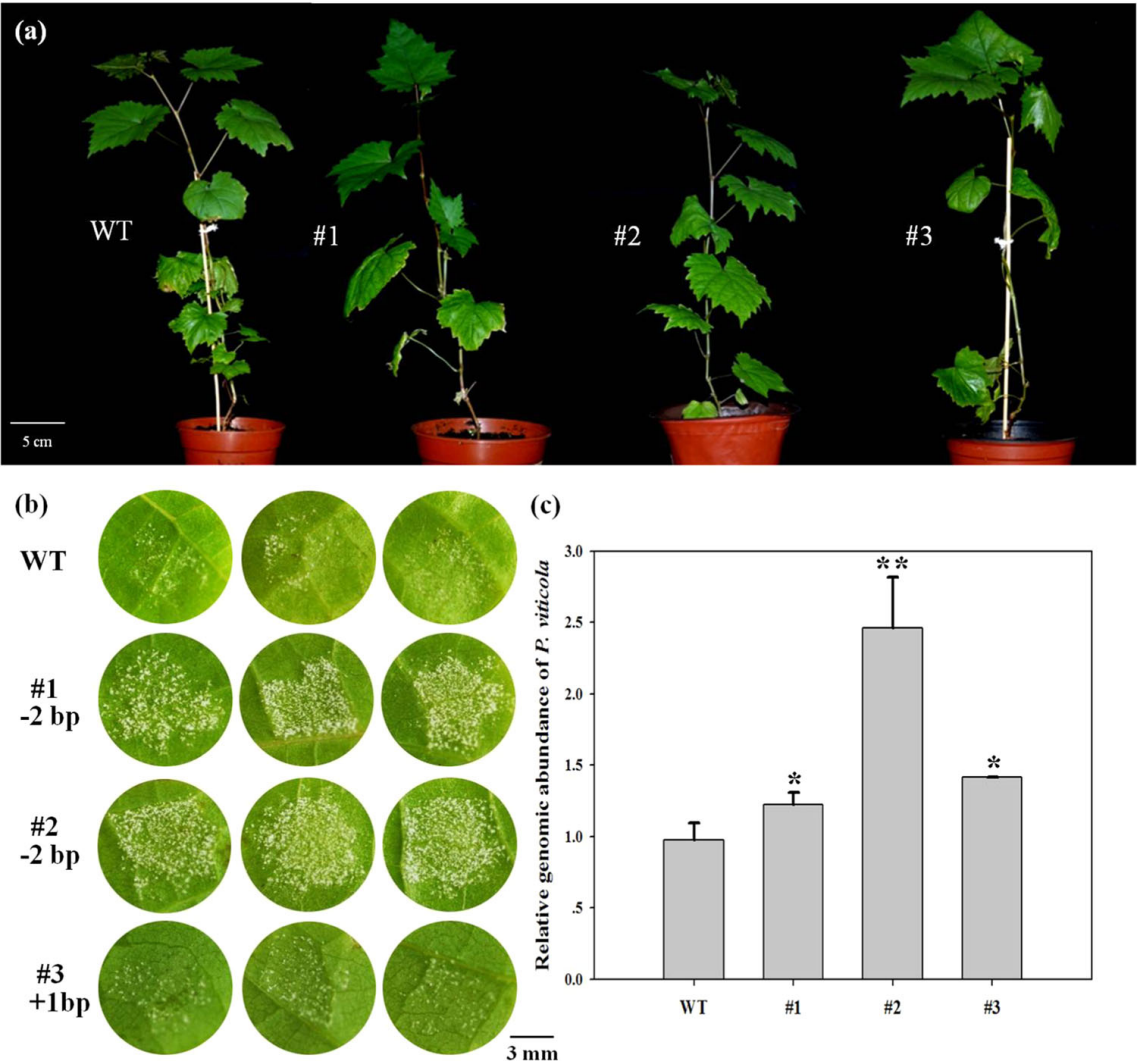
Downy mildew disease progression on leaf discs from wild-type (WT) and *VvPR4b* knockout (#1-3) lines. **a** Phenotype of wild-type (WT) and *VvPR4b* knockout (#1-3) lines in the absence of pathogen; (**b**) phenotype of WT and *VvPR4b* knockout transgenic lines infected with *P. viticola*. Leaf discs were photographed at 96 hpi. Lines #1 and #2 represent the dinucleotide deletion (−2 bp), and line #3 represents the single-nucleotide insertion mutation (+1bp); (**c**) relative genomic abundance of *P. viticola* at 120 hpi. Error bars represent standard errors. An asterisks indicate statistically significant differences between WT and transgenic lines (*P < 0.05, **P < 0.01)

Three representative transgenic lines (#1-3) were chosen to assess the impact of loss of function of *VvPR4b* on the resistance to *P. viticola*. Lines #1 and #2 represented the dinucleotide deletion (CGCCA > CCA), whereas line #3 represented the single-nucleotide insertion (CGCCA > CGCCCA). Under optimal growth conditions and in the absence of the pathogen, there were no visible differences in phenotype between *VvPR4b* knockout lines and wild-type plants (Fig. 4a). We evaluated resistance to *P. viticola* using a detached leaf disc assay. Leaf discs were excised from sterilized leaves of transgenic or wild-type plants, inoculated with a *P. viticola* sporangium suspension, and maintained in petri dishes under 100% relative humidity in a controlled-environment chamber. After 72 h (72 h post-inoculation or hpi), discs from two of the transgenic lines (#1, #2) began to show abundant sporangia, while discs from transgenic line #3 and wild-type plants were relatively unaffected (data not shown). After four days (96 hpi), leaf discs from transgenic lines #1 and #2 were covered with sporangia, while the symptoms on the discs from transgenic line #3 and wild-type plants were much less severe (Fig. 4b). To quantify the relative extent of downy mildew disease progression, quantitative real-time PCR was conducted with oligonucleotide primers specific for *ACTIN* sequences in the grapevine (*VvACTIN1*) or *P. viticola* (*PvACTIN*) genomes. The relative genomic abundance of *P. viticola* in transgenic lines #1, #2, and #3 was 1.2, 2.5, and 1.4 times that in the wild type, respectively, which was consistent with the observed disease phenotype (Fig. 4c).

### Observation of *P. viticola* colonization and reactive oxygen species (ROS)

**Fig. 5:**
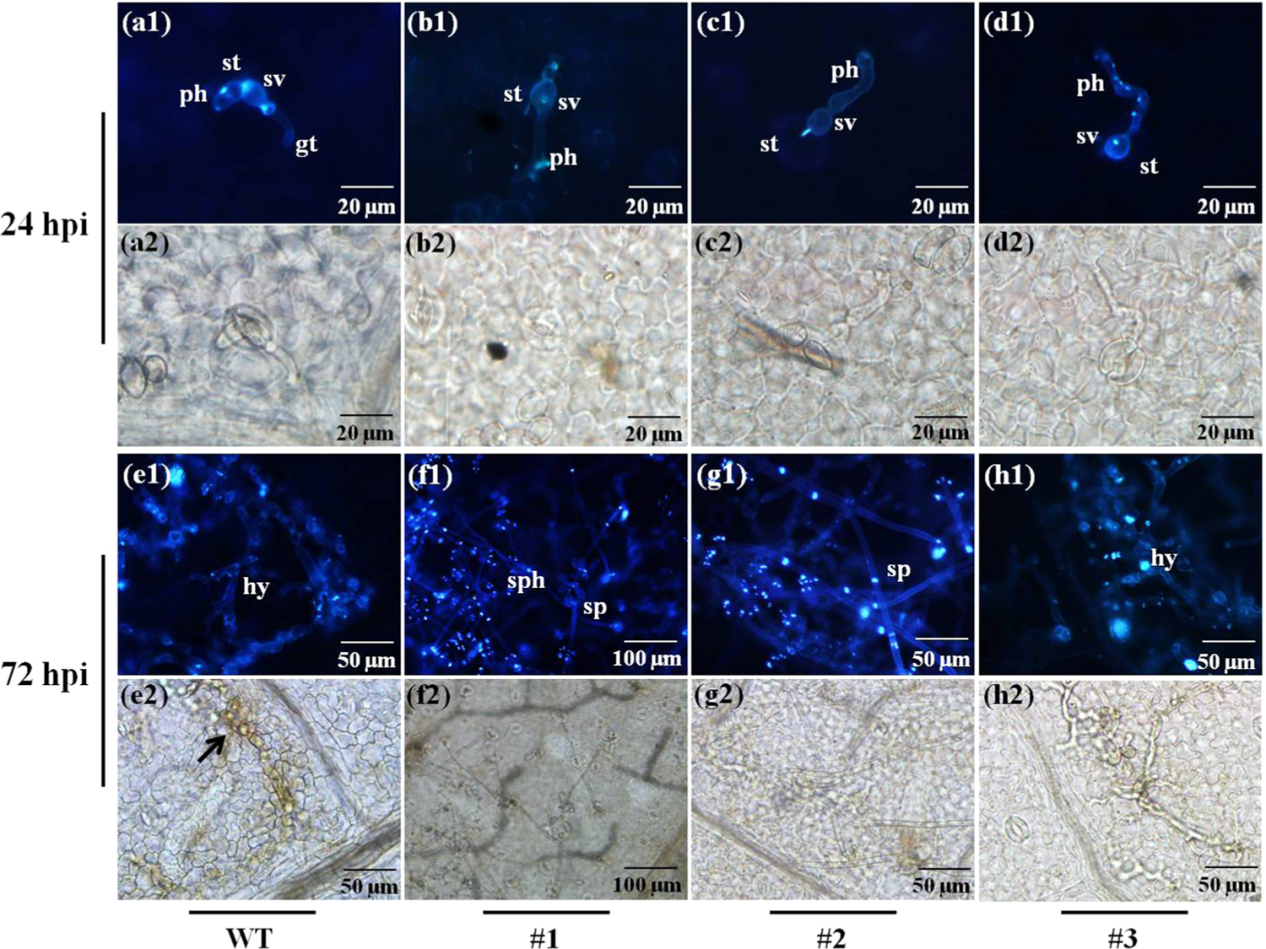
*P. viticola* development and H_2_O_2_ production at inoculation sites in leaf discs of WT and *VvPR4b* knockout lines as revealed by staining with aniline blue and DAB. **a**–**d** and **e**–**f** represent 24 hpi and 72 hpi, respectively. **a1**–**h1** show infected stomata stained with aniline blue under epifluorescence UV-microscopy. **a2**–**h2** show the tissues stained with DAB under bright-field illumination in the field of vision corresponding to (**a1**–**h1**). Arrowhead, H_2_O_2_ accumulation; gt germ tube, st stomata, sv substomatal vesicle, ph primary hyphae, hy hyphae, sph sporangiophores, sp sporangia

To assess downy mildew disease progression within the leaf tissues, we used epifluorescence UV microscopy to examine *P. viticola* colonization. Detailed observations were made at 24 hpi and 72 hpi in both WT and *VvPR4b* knockout lines (Fig. 5). The first sign of infection was the appearance of substomatal vesicles around stomata, which was evident in both WT and *VvPR4b* knockout lines at 12 hpi and 24 hpi (Fig. 5a–d and Fig. S2A1-D1). In addition, germ tubes were observed in the wild type, but not in the *VvPR4b* knockout lines, at 24 hpi (Fig. 5a1). In addition, the mycelia in the WT cells were shorter than those in the three *VvPR4b* knockout lines at 24 hpi (Fig. 5a1–d1 and Fig. S2A2–D2). Finally, staining of cells with 3,3-diaminobenzidine (DAB) failed to detect H_2_O_2_ in either the WT or the *VvPR4b* knockout lines (Fig. 5a2–d2 and Fig. S2a1–d1, a2–d2). By 48 hpi, the mycelia branched rapidly, completely covering the infected area (Fig. S2A3-D3). At 72 hpi, hyphae were apparent in the inner tissues in the wild type and transgenic line #3, but there were no obvious sporangiophores or sporangia (Fig. 5e1, h1). In contrast, we observed abundant sporangiophores and sporangia in *VvPR4b* knockout line #1 (Fig. 5f1). Finally, both the WT and *VvPR4b* knockout lines developed sporangiophores at the inoculation sites at 96 hpi (Fig. S2A5-D5, A6-D6). DAB staining revealed H_2_O_2_ accumulation in the mesophyll tissue of the WT, but staining was generally absent in the mesophyll and was restricted to the periphery of the stomata in the *VvPR4b* knockout lines (Fig. 5e2–g2). Specifically, in the leaves of wild-type plants, DAB staining appeared at 48 hpi around the infection sites, as the mycelia extended and sporangiophores differentiated in the mesophyll tissue with increasing infection time (Fig. S2a1-a6). In comparison, DAB staining at 48 hpi was restricted to the periphery of only a few stomata of transgenic lines #1 and #2 (Fig. S2b1–b6, c1–c6). For transgenic line #3, there was almost no accumulation of H_2_O_2_ throughout the infection process (Figs. 5h2 and S2).

## Discussion

Grapevine is one of the most economically valuable horticultural crops in the world, but increasingly variable biotic and abiotic pressures pose serious threats to grape production. One of the most formidable and widespread diseases of grapevine is downy mildew. Although there have been many studies on the pathology of the causative agent, *P. viticola*, few studies related to functional analyses of resistance genes in grapevine have been investigated. One reason for this is the recalcitrance of grapevine to *Agrobacterium*-mediated transformation, and subsequent regeneration. However, recent advances in transformation technologies using embryogenic callus cells have now overcome this roadblock^28^. Previous approaches to gene targeting in plants have used transcription activator-like effector nucleases and zinc-finger nucleases. These approaches have often been successful but are complicated in target design^29^. The CRISPR/Cas9 system thus represents a tremendous advancement for studies of gene function in grapevine.

In the present study, we used sgRNA to target three independent sites in *VvPR4b* exons. Analyses of mutation frequency and off-target sequences showed that the Cas9/sgRNA expression vector could effectively induce mutations at precise loci in grapevine. Interestingly, we found that the Cas9/sgRNA construct resulted in both deletions and insertions 3 bp upstream of the PAM sequence (Fig. 3b), which was consistent with previous studies^30,31^. Most CRISPR-induced mutations identified in previous studies in grapevine were short deletions and insertions^23–25,27^. However, long deletions (>5 bp) have also been observed, possibly resulting from multiple, closely spaced targets^32^. In our study, the *VvPR4b* knockout lines showed only two mutations that changed the length of *VvPR4b*, namely, a dinucleotide deletion (CGCCA > CCA) and a single C nucleotide insertion (CGCCA > CGCCCA), all of which were located 3 bp upstream of the PAM. Importantly, the base insertion mutations occurred only once in the transgenic lines, whereas the remaining mutations were all two-base deletions (Fig. 3b). We speculate that reason for the low number of mutation types may be related to there being a single target and to the different materials used for genetic transformation (“Chardonnay”/“Thompson Seedless”), but additional experiments are needed to verify this hypothesis. Previous studies have shown that editing efficiency is impacted by GC content in the sgRNA. For example, a sgRNA sequence with >50% GC resulted in higher editing efficiency as compared with a sgRNA with <35% GC^18,23^. In our study, 129 regenerated grape seedlings were identified, of which 26 lines were *VvPR4b* knockout lines, and the gene editing efficiency was 20.16%, with 60% GC content in the target sequence. Furthermore, no off-target mutation was detected in this study (Table 1), which could be due to the use of only a single sgRNA. This suggested that the CRISPR/Cas9 system can be effective in precisely editing genes in grapevine.

However, the mutations found in our study indicated that the allele had undergone distinct editing. For example, (1) a dinucleotide deletion (CGCCA > CCA) was present in allele I, while allele II exhibited base replacement (CGCCA > TAACC), and (2) a single C nucleotide was inserted into allele I (CGCCA > CGCCCA) while allele II exhibited base substitution (CGCCA > TAACC) (Fig. 3c). This gene knockout pattern has been widely observed in previous studies and is expressed as +n/–n (or −n/+n)^23,24^. The protein encoded by these mutations is expected to lack a complete Barwin domain and thus is likely to be dysfunctional (Fig. 3d).

Both the PEM callus and SEs have been found to be suitable tissues for transformation via cocultivation with *Agrobacterium*. Many previous studies have been conducted to improve both transformation efficiency and regeneration in grapevine^33^. In this study, we used PEM from the cultivar ‘Thompson Seedless’, developed through recurrent cycles of secondary embryogenesis (Fig. 2a). In addition, we evaluated the expression of the Cas9 protein in transgenic lines by western blotting. It has been reported that Cas9 remains bound to target DNA after cleavage^25,34^.

It is well known that PR proteins play pivotal roles in plant defense against pathogen challenge. PR4, which is a chitinase and chitin-binding protein, plays an important role in pathogen responses in many plants. In this study, we demonstrated that *VvPR4b* encodes a chitinase II-like protein containing a amino-terminal signal peptide and carboxyl-terminal Barwin domain (Fig. 1).

A previous study showed that the expression of *PR4* was induced after inoculation in ‘Riesling’, suggesting that PR4 may play a role in the resistance to *P. viticola*^35^. In our study, at both 72 hpi and 96 hpi there was more extensive colonization on leaf discs from two *VvPR4b* knockout lines (#1 and #2), than on discs from the WT (Fig. 4b). We also found that at 120 hpi, the relative genomic abundance of *P. viticola* in all three *VvPR4b* knockout lines was obviously higher than that in the WT (Fig. 4c), which demonstrated that *VvPR4b* is required for full resistance to *P. viticola*.

Leaf discs stained with aniline blue 12, 24, 48, 72, 96, and 120 hpi (Fig. S2) were used to further evaluate colonization by *P. viticola*. The infection cycle of *P. viticola* includes the attachment of the zoospore to a stoma and generation of a germ tube, which enters the substomatal cavity and expands into a substomatal vesicle. Subsequently, hyphae are differentiated and spread into the intercellular space of the parenchyma^36^. At 96 hpi, we observed that the inoculation sites of the WT and *VvPR4b* knockout lines were full of sporangiophores (Fig. S2A5-D5, A6-D6), which because that “Thompson Seedless” has relatively low resistance to grapevine downy mildew. Meanwhile, the growth of *P. viticola* was consistent with the phenotype of grape leaf discs infected with downy mildew (Fig. 4), reflecting the difference in resistance between wild-type and *VvPR4b* knockout lines.

The mechanism underlying the plant response to fungal infection, and how this affects the interaction between plants and pathogens, involves a very complex regulatory network in which the generation of ROS plays an important role, including in the regulation of gene expression of PR1, chitinase and β-1, 3-glucanase^37^. It has been reported that the PR4b protein has antifungal activity in pepper (*Capsicum annuum*), wheat (*Triticum aestivum*) and rice (*Oryza sativa* L.)^38^. In tobacco, the *PR4* gene was induced by overexpression of *SWPA4* (a major stress-induced gene), resulting in increased H_2_O_2_ production^39^. PR1, PR4, and PR10 were significantly upregulated in OsWRKY67-overexpressing transgenic lines, with rapidly accumulation of ROS to increase the resistance to two rice pathogens (*Magnaporthe oryzae* and *Xanthomonas oryzae* pv. *oryzae*)^40^. Similarly, the relative transcriptional level of PR genes was upregulated in *OsMAPK15* (a negative regulatory gene for disease resistance in rice) knockout mutants, while chitin promoted an increase in the accumulation of ROS and significantly enhanced the resistance of rice to the two abovementioned diseases, and the opposite results were obtained in these gene overexpression lines^41^. In our study, we used DAB staining to visualize the accumulation of H_2_O_2_ inside the leaf tissues (Fig. S2). H_2_O_2_ is produced in cells as a defensive response to pathogens that infect via stomata^42,43^. Previous studies have shown that both susceptible and resistant cultivars of grapevine produce ROS when infected with downy mildew^44^. Our DAB staining results suggested that *P. viticola* developed rapidly in the *VvPR4b* knockout lines compared with the WT.

Overall, our research demonstrated that the CRISPR/Cas9 system can be a high-efficiency tool for generating targeted mutations in grapevine and will be beneficial for gene functional analyses in this plant. In addition, this work showed that grapevine *VvPR4b* plays an important role in downy mildew resistance.

## Materials and methods

### Plant materials

Proembryogenic mass callus cells induced from immature anthers of “Thompson Seedless” were used for transformation according to previously published protocols^11,12^. The induced PEMs were recultured in KBN medium (Murashige-Skoog basal medium (M519) containing 30 g/L sucrose, 1 g/L inositol, 0.3 g/L KNO_3_, 1.126 mg/L 6-BA, 1.104 mg/L 2,4-D, 1.012 mg/L NOA, and 3 g/L phytagel; pH 5.8). “Thompson Seedless” PEM was propagated on KBN and X6 (Murashige-Skoog basal medium containing 60 g/L sucrose, 3.0 g/L phytagel, and 1.5 g/L activated carbon; pH 5.8), changing to fresh medium once a month. The PEM was cultured in darkness at 24–25 °C.

### Amplification of the *VvPR4b* genomic clone

The *PR4* gene in “Pinot Noir” is located on chromosome 14, has a total length of 527 bp, and contains a 95-bp intron (Grape Genome Browser 12x; http://www.genoscope.cns.fr/externe/GenomeBrowser/Vitis/). This sequence was used to design oligonucleotide primers for the amplification of *VvPR4b* from “Thompson Seedless” (Table S1). For amplification, we employed a high-fidelity DNA polymerase (KOD-plus Neo, KOD-401(856600), TOYOBO, Japan) in a total reaction volume of 50 μL. The amplification parameters were 94 °C for 3 min; 40 cycles of 98 °C for 10 s, 59 °C for 30 s, and 68 °C for 25 s; and a final extension at 68 °C for 7 min. The PCR product was cloned into the pLB-Simple vector and introduced into the *E. coli* strain Top10 for propagation and subsequent sequencing.

### Target selection and CRISPR/Cas9 knockout vector construction

To identify potential target sites in *VvPR4b*, we analyzed the entire *VvPR4b* genomic sequence using CRISPR-P (http://cbi.hzau.edu.cn/cgi-bin/CRISPR). sgRNAs were selected on the basis of their position in the gene and the potential for off-target mutations. To generate the binary vector, the pBR322-based pCACRISPR/Cas9 vector was subjected to recombination with the VvPR4b-AtU6-sgRNA cassette.

### Grapevine transformation and regeneration

The PEM was cultured for 7 days prior to transformation to improve activity on culture medium X6. The *VvPR4b* CRISPR/Cas9 binary vector was transformed into *Agrobacterium tumefaciens* strain GV3101 through the freeze-thaw method^45^. *Agrobacterium* was grown in LB liquid medium containing antibiotics [gentamicin (Gent), rifampicin (Rif), and kanamycin (Kan)]. Cells were collected by centrifugation and resuspended in resuspension solution (½-strength MS liquid medium containing 100 μmol/L acetosyringone). The infection procedure was carried out as described by Su^46^ with minor changes. Briefly, the resuspended culture was incubated on a shaker at 28 °C for 30 min, the density was adjusted to OD600 = 0.75, and the PEM callus was added. The combined *Agrobacterium*/PEM culture was then incubated for 10 min with shaking. Then, PEM cells were collected on sterile filter paper soaked in resuspension solution and cultured in darkness at 24 °C for 48 h. Then, the PEM was washed with sterile water containing 500 mg/L cefomycin (Cef) and 500 mg/L carbenicillin (Carb), transferred to delayed screening medium (Murashige-Skoog basal medium containing 60 g/L sucrose, 3.0 g/L phytagel, 1.5 g/L activated carbon, 250 mg/L Cef, and 250 mg/L Carb; pH 5.8) and cultured in darkness at 24 °C for 1 month. Next, the PEM was subcultured on X6 screening medium (Murashige-Skoog basal medium containing 60 g/L sucrose, 3.0 g/L phytagel, 1.5 g/L activated carbon, 250 mg/L Cef, 250 mg/L Carb, and 7.5 mg/L hygromycin (Hyg); pH 5.8). The nontransformed tissue gradually died and turned black. The resistant embryos were transferred to X3 screening medium (Murashige-Skoog basal medium containing 30 g/L sucrose, 3.0 g/L phytagel, 1.5 g/L activated carbon, 250 mg/L Cef, 250 mg/L Carb, and 7.5 mg/L Hyg; pH 5.8) to promote development into plantlets. After cotyledon formation, plantlets were transferred to X1.5 (Murashige-Skoog basal medium supplemented with 15 g/L sucrose, 3.0 g/L phytagel, and 1.5 g/L activated carbon; pH 5.8) and maintained under a 16-h light/8-h dark photoperiod to induce germination. Then, the long embryonic axis was transferred to WPM medium (WPM basal medium containing 30 g/L sucrose, 7.0 g/L agar, and 0.2 mg/L 6-benzyladenine (6-BA)) to regenerate the plant^46^. Finally, the regenerated seedlings were transplanted into an artificial soil mixture and cultured in a controlled-environment chamber.

### Identification of transgenic lines

To identify regenerated plants containing the *VvPR4b*-CRISPR binary vector, young-leaf genomic DNA was extracted through a CTAB-based method and subjected to amplification using oligonucleotide primers specific for the RTcas9 and Hygjc2 sequences (Table S1). The *VvPR4b* target site sequence was amplified by PCR using a high-fidelity DNA polymerase (KOD-plus Neo, KOD-401(856600), TOYOBO, Japan) using the cri-pr4b-F/R primers (Table S1) and sequenced with the specific primer cri-pr4b-cx (Table S1). In addition, the PCR products were extracted from the gel and cloned into the pMD19-T vector (code no.6013, TAKARA). For each transgenic line, 10 randomly selected clones were sequenced. Sequences were aligned by DNAMAN (version 6.0.40; Lynnon Biosoft).

### Western blotting

For analysis of Cas9 protein expression in transgenic plants as shown in Fig. 4, total proteins were extracted from leaves by homogenization with PPEB extraction buffer (0.1 M Tris-HCl, 2% SDS, 10% glycerin, 0.05 M β-mercaptoethanol). The extracted proteins were subjected to SDS-polyacrylamide gel electrophoresis (SDS-PAGE) and transferred to polyvinylidene fluoride membranes by electrophoresis. The membranes were incubated overnight at 4 °C in Tris-buffered saline with Tween (TBST; 0.02 M Tris-HCl (pH 7.4), 0.15 M NaCl, 0.05% Tween 20) containing mouse anti-FLAG antibodies (1: 4000, AE005, ABclonal). Subsequently, membranes were washed in TBST, and incubated with HRP-labeled goat anti-mouse IgG (H + L) (1: 10000, DY60203, DIYIbio) at room temperature for 2 h. After washing in TBST, the membrane was processed with the Ultra-Sensitive ECL Chemiluminescence Kit (P0018AS, Beyotime) and was then observed and photographed with a Biorad imager (Biorad ChemiDoc MP)^47^.

### Off-target analysis

Putative off-target sites were identified through CRISPR-P. Oligonucleotide primers specific for the sites (Table S1) were used for PCR amplification with high-fidelity DNA polymerase (KOD-plus Neo, KOD-401(856600), TOYOBO, Japan). Amplified sequences were cloned into the pMD19-T vector (code no.6013, TAKARA) for sequencing.

### Pathogen culture and inoculation

*P. viticola* was isolated from an infected leaf of a grapevine plant^48^ in the Grape Repository of Northwest A&F University, Yangling, Shaanxi, China. The leaf was washed three times aseptically, placed with the abaxial side facing upwards on wet filter paper, and cultured overnight in darkness at 22–25 °C. The following day, zoospores were collected in sterile water with a sterilized soft brush, and the suspension was agitated in the dark for 10 min and then filtered through three layers of gauze. Sporangia were counted using a hemocytometer, and the concentration was adjusted to 5 × 10^4^ per mL in sterile water. For leaf disc assays, leaves were detached from plants maintained in a controlled-environment chamber, washed three times with sterile water, and allowed to dry at room temperature. Discs were excised using a 10-mm-diameter, stainless steel hole punch and placed with the abaxial surface facing upwards on three layers of sterile wet filter paper in a petri dish. Then, 20 μL of the sporangial suspension was applied, and samples were incubated for 24 h in darkness at 22 ± 2 °C with 100% RH, followed by incubation at the same temperature under a 16-h light/8-h photoperiod. Experiments were carried out with three biological replicates for each line (WT or different transgenic lines), with each replicate using at least nine leaf discs^49,50^.

### Statistics on the relative genomic abundance of *P. viticola*

To determine the relative genomic abundance of *P. viticola* infecting leaf discs, the inoculated discs were collected. Genomic DNA was extracted using a CTAB-based method, and the DNA concentration was adjusted to 50 μg/L. For PCR amplification, 4 μL of genomic DNA was used in combination with oligonucleotide primers specific for the *VvACTIN1* and *PvACTIN* genes (Table S1). The relative genomic abundance of *P. viticola* was calculated as 2^−▵▵Cq 51,52^.

### DAB and aniline blue staining and analyses

For detection of H_2_O_2_ using DAB, leaf discs were immersed in 1 mg/mL 3, 3-diaminobenzidine solution (DAB, Sigma, dissolved in distilled water at pH 3.8, acidified by hydrochloric acid) and then cultured under light for 8 h. The DAB staining solution was then replaced with 95% alcohol, and the samples were decolorized in saturated chloral hydrate solution (250 mg/100 mL) until translucent, washed three times with 0.05% aniline blue (dissolved in 0.067 M K_2_HPO_4_, pH 9-10) and incubated overnight in the same solution. The leaf discs were placed on a glass slide with the abaxial side facing up, and hyphal growth was observed under blue/purple light with a fluorescence microscope (Olympus bx-51)^49,53^. The accumulation of H_2_O_2_ was observed under a bright field.

### Data analysis

Data were analyzed by SPSS 16.0 software, and significant differences were identified using the Tukey test for one-way ANOVA.

## Supplementary information

Supplemental Materials
