# Supplementary material for: CRISPR/Cas9-mediated VvPR4b editing decreases downy mildew resistance in grapevine (Vitis vinifera L.)
Source: Hortic Res. 2020 Sep 1;7:149. doi: 10.1038/s41438-020-00371-4 (PMC7458914; doi:10.1038/s41438-020-00371-4)
Supplement: Supplementary file 1 — Supplemental Materials [file 41438_2020_371_MOESM1_ESM.doc]

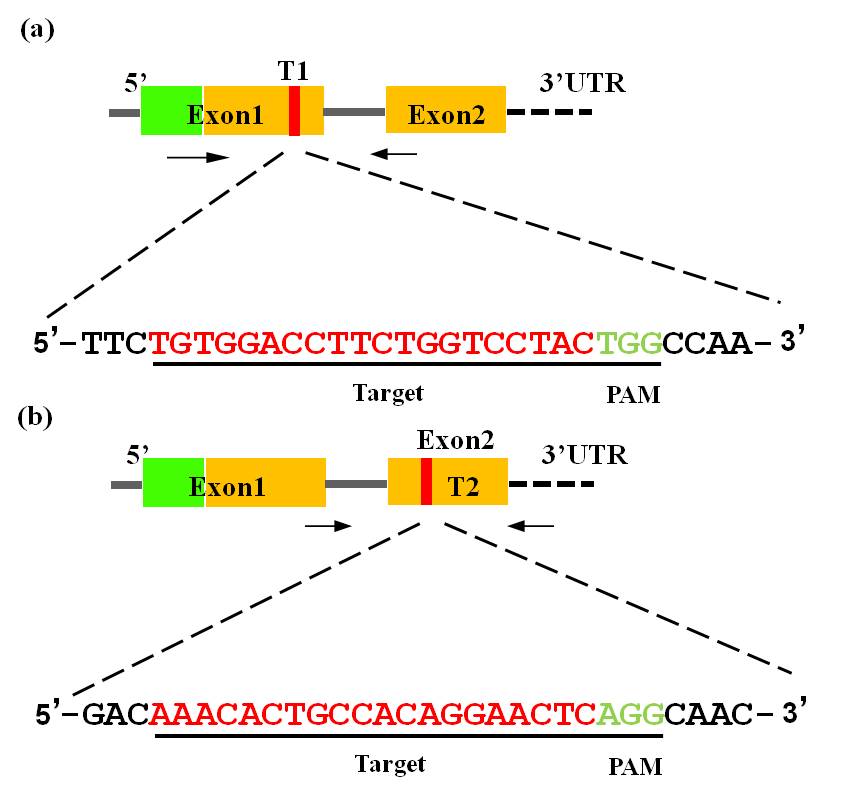


**Fig. S1** Schematic of the location of other two target sites. Exons are shown as green and yellow blocks, where green indicates the signal peptide and yellow indicates the Barwin domain. Target sites are indicated by vertical red bars. The expanded sequence for target (T1 or T2) includes the target sequence (red) and PAM trinucleotide (green).


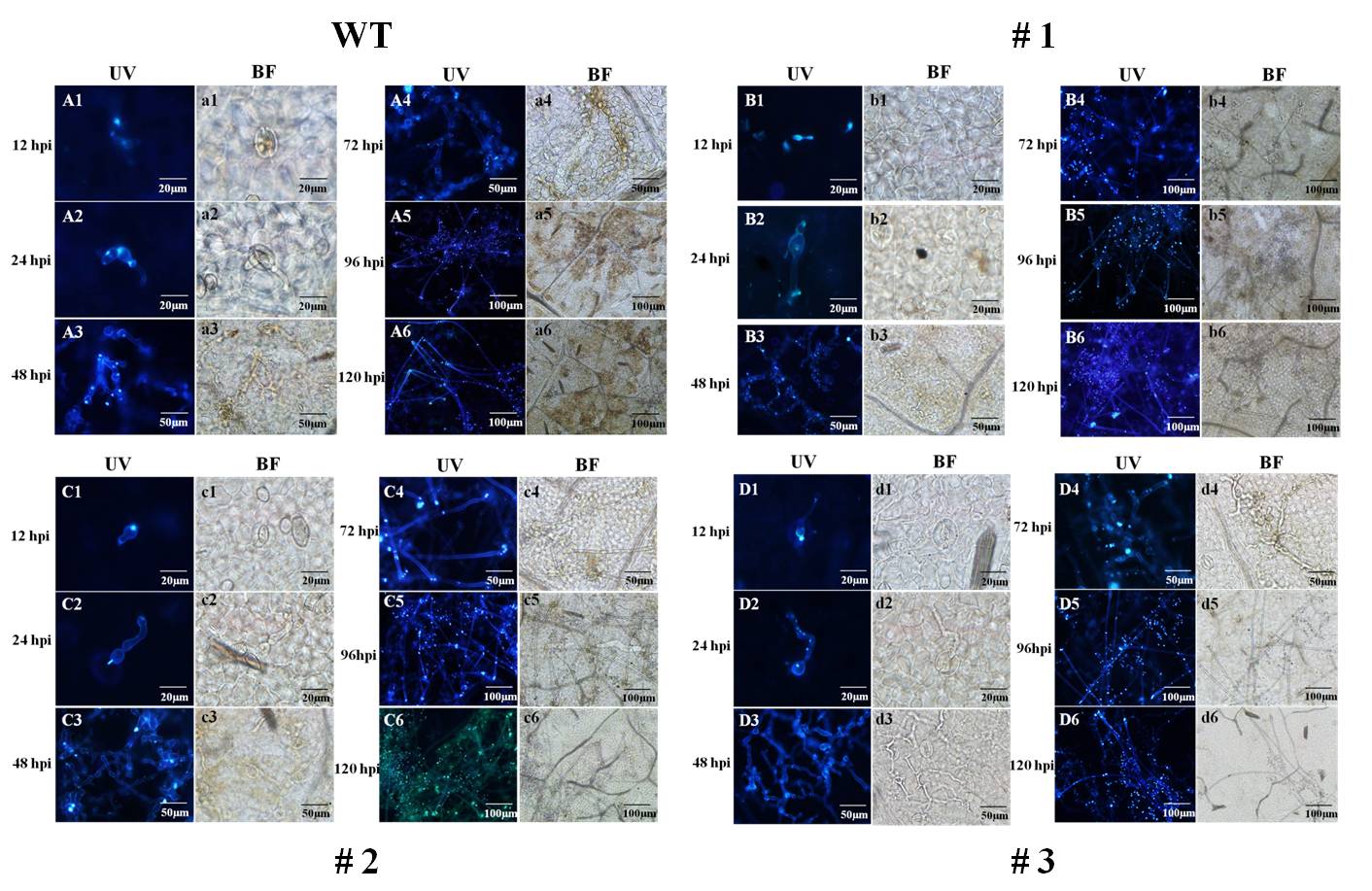


**Fig. S2** Aniline blue staining and H_2_O_2_ production in *VvPR4b* knockout lines inoculated with *P*. *viticola*. Infected stomata with *P*. *viticola* at times after inoculation (12, 24, 48, 72, 96, 120 hpi) visualized by staining with aniline blue and epifluorescence UV-microscopy (A-D); H_2_O_2_ accumulation visualized by staining with DAB under bright field (BF) illumination (a-d).

**Table S1. Primers in this study**

| Primer name | Primer sequence (5’→3’) | Use |
| --- | --- | --- |
| VvPR4b-F | ATGGAGAGGAGAGGCATATGCAAGG | Vector construction |
| VvPR4b-R | GTCACCACAGTTCACAAACTGGTAATTGA |
| U6p.4-F | CAGGAAACAGCTATGACCATATTCATTCGGAGTTTTTGTATC |
| Oligo-R | GCTATTTCTAGCTCTAAAAC-TTGGCGTAGCCAGCCCCATTC-  AATCACTACTTCGACTCT |
| RTcas9-F | AAGCCCATCAGAGAGCAGG | Detection of transgenic lines |
| RTcas9-R | TGTCGCCTCCCAGCTGAG |
| Hygjc2-F | GTCCGTCAGGACATTGTTGGAGCC |
| Hygjc2-R | GTCTCCGACCTGATGCAGCTCTCGG |
| cri-pr4b-F | ATGGAGAGGAGAGGCATATG |
| cri-pr4b-R | GCAGTAGAAGCGGCTAACTCCATG |
| cri-pr4b-cx | GAACGCAGTGAGCGCCTACTG |
| Chr14-F | GTGACAAACACTGCCACAGGAACTC | Analysis of off-target |
| Chr14-R | TCGATGGTCACATAACTCATCAGCTAAG |
| Chr16-F | CTCCACGACATGTTGCCTTAACTGC |
| Chr16-R | GCCACGCCCATAGGGCTTTCTTA |
| Chr11-F | GTGGCTTATGTCCTAGCTACTGATTGC |
| Chr11-R | GTGGAAATGACCATGAGTATAAAGCTCCA |
| Pvactin-F | CTCCAGAACGTGTACATCCG | Relative genomic abundance of *P. viticola* |
| Pvactin-R | TAGCGCCCTTCTCCTCAG |
| Vvactin1-F | CCATCCTTCGTCTTGACCTTGCTG |
| Vvactin1-R | AGTGGTGAACATGTAACCCCTCTC |
